# 

**Published:** 1851-07

**Authors:** 


### TO READERS AND CORRESPONDENTS.

Communications intended for publication, and all letters relating to the business of the Journal, or containing remittances in money, should be directed to Drs. Harris and Blandy, Baltimore, Md.

The following Journals and Publications have been received:

The Dublin Quarterly Journal of Medical Science, May, 1851. Nos. 19, 20 and 21, have not been received; we would be glad if the publisher would be kind enough to forward them to us.

The London Medical Gazette, January, February, March and April, 1851.

The Boston Medical and Surgical Journal. Edited by J. V. C. Smith, M. D., April, May and June, 1851.

The British and Foreign Medico-Chirurgical Review, April, 1851.

The New York Dental Recorder. Edited by C. C. Allen, M. D., April and May, 1851.

The Dental Register of the West. Edited by James Taylor, M. D., D. D. S., April, 1851.

The Dental News Letter, April, 1851.

The Buffalo Medical Journal. Edited by Austin Flint, M. D., April, May and June, 1851.

The Provincial Medical and Surgical Journal. Edited by W. H. Rankin, M. D., and J. H. Walsh, Esq., March and April, 1851.

The Charleston Medical Journal and Review. Edited by D. J. Cain, M. D., and F. Peyrel Porcher, M. D., May, 1851.

The New York Journal of Medicine and the Collateral Sciences. Edited by S. S. Purple, M. D., April and May, 1851.

The Medical Examiner. Edited by F. G. Smith, M. D., and John Biddle, M. D., April, May and June, 1851.

The Dublin Medical Press, March, April and May, 1851.

The Southern Medical and Surgical Journal. Edited by J. P. Garvin, M. D., April, May and June, 1851.

The British American Medical and Physical Journal. Edited by Archibald Hall, M. D., November, 1850, April and May, 1851.

The New York Medical Gazette, and Journal of Health. Edited by D. M. Reese, M. D., LL. D., April, May and June, 1851.

The North-Western Medical and Surgical Journal. Edited by J. Evans, M. D., and E. G. Meek, A. M., M. D., May, 1851.

The Ohio Medical and Surgical Journal. Edited by Richard L. Howard, M. D., May, 1851.

The St. Louis Medical and Surgical Journal. Edited by M. L. Linton, M. D., J. S. Moore, M. D., Wm. M. McPheeters, M. D., and J. B. Johnson, M. D., March and April, 1851.

The Western Journal of Medicine and Surgery. Edited by L. P. Yandell, M. D., and T. S. Bell, M. D., April, May and June, 1851.

The New Hampshire Journal of Medicine. Edited by Edward H. Parker, A. M., M. D., April, May and June, 1851.

The New York Register of Medicine and Pharmacy. Edited by C. D. Griswold, M. D., April, May and June, 1851.

The Western Lancet and Hospital Reporter. Edited by L. M. Lawson, M. D., and George Mendenhall, M. D., April, May and June, 1851.

### *To Readers and Correspondents.*

The London Lancet, Journal of British and Foreign Medical, Surgical and Chemical Sciences, Chemistry, Literature and News. Editor, Thos. Wakeley, M. D., for London: Sub-Editors, J. H. Bennet, M. D., and T. Wakeley, M. R. C. S. E., April, May and June, 1851.

The New Jersey Medical Reporter, and Transactions of the New Jersey Medical Society. Edited by Joseph Parish, M. D., March and April, 1851.

The London Medical Times has not been received since November, 1850; will the publishers be good enough to forward the missing Nos.

The Annual Catalogue and Announcement of the Medical Department of the St. Louis University, for 1851 and 1852.

The Proceedings of the Medical Association of the State of Alabama, 1851, from our friend G. Gage, M. D.

Merritt's Dental Messenger, Nos. 1 and 2, published quarterly, 1851.

*A good Operator Wanted.*—We can find a good situation for a competent young man as an operator, in the capacity of assistant; location in the south.

Also, a gentleman, of some capital, can find an excellent opportunity to invest his means, provided he can fill the duties of a good dentist. All necessary information can be had by applying to the Editors of the Am. Journal of Dental Science; *letters post paid.*

Also, a skillful Carver, in block work, can hear of a good situation, by applying, *letters post paid*.

*Articles Received.*—"*Attachment of Springs.*" We should have published this article as the necessary description is admirable, but the difficulty of obtaining proper wood cuts have prevented. Its author must receive this as our apology, with regrets.

We must beg to differ with C. S. B., of L., as regards the proposed use of *chloroform.* We have tried it both before and after the receipt of his article, and have never experienced the results he describes. Persevere and give us your opinion after a few more practical tests.

Many inquiries are made of us concerning the comparative utility of "*block* and *single teeth*," and as answer to all, we submit the following opinion derived from our experience. In block teeth, there is great superiority in the possibility of so imitating nature as to be without a shade of perceptible difference, by giving a natural expression and in renewing the contour of the attenuated gums—thus producing a general and happy effect upon the whole face, and absolutely preventing any accumulations. In single teeth there are many cases in which these points cannot be attained. Their mechanical uniformity being generally such as will not permit the artist to adapt the best selection to the anatomical signatures of the face; all of which can be accurately accommodated by the ingenious block workman; but only for full upper or lower sets. As a general rule we do not approve of partial sets of blocks. It must be remembered, however, that the great improvements within the last few years, in blocks, have removed the objections which previously existed to dental substitutes of this description.

If Dr. K. will give a full description of the case under the head of "*Amalgam Fillings*," we shall be happy to publish, but as it is, we are constrained to say it is incomplete, and its pathology carelessly understood by the writer, who might expose himself to the continuance of the unhappy controversy.

The article on "*Tardy First Dentition*," is a little faulty; will its author re-write with care.

*To A.*—A certain galvanic action must always take place during the presence of two metals in the mouth, which are subjcct to different degrees of oxydation.

*Adjusting Forceps,* came too late for the present No. It will appear in our next, and with suitable wood cuts.

The article intended to illicit information, upon the use and experience of *chloride of zinc,* shall be attended to in No. 1, Vol. 2, October next.

*Some remarks on the operation of filling teeth*—This is very good, but came too late for this No.

*Cases of Cancrum Oris,* by J. L. Levison, Esq., of Brighton, England. This came too late for this No., but will appear in our next. The cases are interesting, and we shall be always happy to receive communications from the same able writer.

*The Auto-Biography of a Tooth.*—From press of business we have not had time to examine it, but doubt not it is good from our knowledge of the ability of its author, to whom we are indebted.

### CONTENTS.

### JOURNAL.

Page.

*Twelfth Annual Meeting of the American Society of Dental Surgeons.*—The twelfth annual meeting of this association will commence its session the first Tuesday, 5th of August, at the United States Hotel, in Philadelphia, and as the place of meeting is quite central, we hope it will be well attended. We would be particularly gratified to meet, on that occasion, the members from the southern and western states. Heretofore they have complained, and we admit with some show of justice, that the meetings have been held too far north, and refused to attend on that account. We trust the place designated for the next meeting, will not be found objectionable to any, and that the members generally will endeavor to be present.

*Completion of the First Volume of the New Series.*—This number completes the first volume of the new series of the Journal, and although we were compelled to strike from our list the names of nearly one-half of the old subscribers, for delinquency, we are gratified to be able to state, that the number has more than doubled during the past year; and although some hundreds are yet in arrears, we sincerely trust, that none will long remain so. We need the money to enable us to carry on and improve the publication, and if those of our subscribers who are in arrears, will be a little more prompt in the payment of their subscriptions, it will save us much trouble and perplexity, and enable us to make the work more valuable than we have hitherto been able to do. In this connection, we would suggest to those who are in arrears, that, in sending the amount now due, it will afford a favorable opportunity of remitting the subscription for the next volume.

*Chevalier's Drill Stock.*—Since writing the notice of this instrument, we have received a very beautifully finished one, and find it useful. For many operations, such an instrument is indispensable.

### INDEX.

PAGE.

A

Adjustment of Clasps, . 31
Artificial Teeth and Plates, 32
Am. Soc. Dent. Surgeons, ninth annual meeting of, . . . 36
American Society of Dental Surgeons, report of committee on rescinding Amalgam resolutions, . . . 66
Antrum, diseases of the, . 79
Am. Soc. Dent. Surgeons, eleventh meeting of, . . . 91, 99
Artificial Teeth in a base of Block Tin, . . . . 103
Application of Gutta Percha to dental purposes, . . . 145
Antrum, successful operation on, . . . . . . . 161
Antrum, operation on, . 169
Artificial Teeth on atmospheric principle, . . . . 190
Advertising Dentists, cheap, 224
Adjuster for Fractures of the Lower Jaw, Reinhardt's, . 226
Arthur, Dr. Robert, on treatment of Dental Caries complicated with Affections of the Pulp, . . . 229, 405
Artificial Teeth, cheap, and cheap Dental Operations in England, . . . . . 240
Artificial Palate, new kind of, with observations on the changes which take place in the Velum and Pharynx, during Deglutition, Respiration and Articulation. By Dr. S. P. Hullihen, . . . . 245
Atmospheric Pressure. By Jas. Flemming, M. D. . . . 308
Acidulated Tooth Powders, 401

PAGE.

Appearance, on the, of the Gums in Phthisis, . . . 372
American Philosophical Society, proceedings of the, April, December, 1850, . . . 394
Addington, R. D., D. D. S., Operations on the Antrum, 169
Address, Valedictory, . . 460
Antrum, diseases of the, . 497
Antrum, collapse of, &c. . 547
Arsenious Acid, by, &c. . 549
Antidote to Arsenic, . . 562
Am. Soc. of Dent. Surgeons, twelfth meeting of the, . 564

B

Base for Artificial Teeth, on the use of Tin as a, . . . 3
Blow-pipe and Lamp, Zeiderholz's Self-acting, . . 103
Bone and Teeth, a piece of, . 218
Biographical Notice of the late Dr. James Gardette, . 375
Baltimore College of Dental Surgery, commencement of the, . . . . . 403
Bond, Thos. E., M. D., a practical Treatise on Dental Medicine, . . . . 209
Blandy, Alfred A., M. D., on the Sensibility of the Teeth, 22
Bell, John, M. D., an Introductory Lecture by, . . . 393
Beaumont, Prof. W. R., Disarticulation of Lower Jaw, 83
Buckingham, T. L., Mechanical Dentistry, . . . 305
Beckett, Chas., M. R. C. S., a few Practical Observations on Diet, especially, that of children and invalids, . . 386

PAGE.

C.

Clasps, Adjustment of, . 31
Clasps for partial sets of Artificial Teeth, . . . . 488
Committee, Report of Am. Soc. of Dent. Surg. on the propriety of rescinding the Amalgam Pledge, . . . 66
Chemistry and Vegetable Food, 87
College, Ohio, of Dental Surg. 100
Correction, . . . . 104
Cleaning Plates after Soldering, 107
Cheap Advertising Dentists, 224
Chairs, &c. . . . . 226
Caries, treatment of Dental, by Robert Arthur, . . . 229
Cheap Artificial Teeth, 240, 441
Cushman, C. T., a Test of Levitt's patent Enamel, . 263
Cancerous Tumors within the Antrum, . . . . . 312
Chloroform, Fatal Results, &c. 315
Chloroform and Ether, their Employment in, &c. . 396
Commencement, annual, of Balt. College Dent. Surg. 403
Corundum Wheels, . . . 414
Chevalier, John D., . . . 404
Crane, J. W., M. D., on wide and narrow Plates for Artificial Teeth, . . . . 458
Chloroform, nine thousand cases, . . . . . 529
Chloroform, New Method of Remedying Accidents caused by, . . . . . . 539
Caries of the petrous portion of the Temporal Bone, . 548
Castle, A. C., M. D., on the Use and Abuse of Arsenious Acid, . . . . . 549
Chloroform, on the Administration of, . . . . . 551
Complimentary Letters, 562
Compound Lever Forceps, 562
Completion of First Volume of new series, . . . . 564
Chevalier's improved Drill Stock, . . . . . 564

D

Dental Organs, relation between Uterus and the. By Prof. W. R. Handy, M. D. 127

PAGE.

Dentists, Percentage System amongst, . . . . 151
Disease of Antrum Highmorianum of the right side. By Chas. H. Dubs, Dentist, . 161
Dentistry, Mechanical. By T. L. Buckingham, dentist, 174, 305
Dental Surgery, Progress of, 222
Dentists, Cheap Advertising—extraordinary Verdict, . 224
Drill Stock, another, . . 225
Dental Instruments—to Inventors, . . . . . 226
Dental Caries, Treatment of, complicated with Affections of the Pulp. By Robert Arthur, D. D. S., . . 229
Dental Operations, Cheap, in England, and Cheap Artificial Teeth, . . . 240
Daily Jottings in the Gold Book of a Dentist, January 1 to March 1. By E. Townsend, D. D. S., No. 1, . . 263
Dental Pulp, Treatment of, preparatory to Plugging. By J. D. White, Dentist, 297
Diseases of the Inferior Maxilla, delivered by John Simon, Esq., . . . . . 332
Displacement of the Epiglottis. By Jas. Ritsell, Esq. . 374
Douloureux, Tic, and other painful Affections of the Nerves, with suggestions for their Treatment by means of the Aneuralgicon. By Toogood Downing, M. D. . 386
Diet, a few Practical Observations on. By Chas. Beckett, . . . . 386
Diseases and Surgical Operations of the Mouth and parts Adjacent, with notes of interesting cases, Ancient and Modern. By M. Jourdain, . . 391
Drake, Daniel, M. D., a Systematic Treatise, Historical, Etiological and Practical, on the principal Diseases of the Interior Valley of North America, by, . . . . 392
Dental Colleges, the propriety

PAGE.
of Abolishing and Substituting Lectureship in Medical Schools, . . . . . 395
Dubouchet, C. A., M. D., on Clasps for partial Sets of Artificial Teeth, . . . . 488
Drew, Joseph, M. B. . . . 532
Dislocation of the Lower Jaw, 554
Dental Medicine, a Practical Treatise on. By Thos. E. Bond, M. D, 209, 269, 387
Dentists, the Touting System, amongst, . . . . 16
Dental Pulp, Treatment of Exposed. By H. K. Nutz, 35
Dental Surgeons, ninth annual meeting of, . . . . 36
Dental Surgeons, report of committee of, . . . . 66
Disease of the Antrum, . 79
Disarticulation, case of, of the Left Condyle of the Lower Jaw. By W. R. Beaumont, 83
Deafness, Partial, of twenty years standing, . . . 89
Dislocation of the Lower Jaw, new method of Reducing, 90
Dental Surgeons, eleventh annual meeting of, . . . 91
Dental Surgeons, Am. Soc. of, 99
Dental Surgery, Ohio College of 100
Drill Stock, new, . . . 191
Dental Surgery, Transylvania School of, . . . 107
Dental Files, . . . . 108
Dental Neuralgia. By A. C. Dayton, M. D., . . . 109
Dubs, Chas. H., successful Operation on, and Treatment of, Antrum Highmorianum, 161
Dayton, A. C., M. D., on Dental Neuralgia, . . . . 109
Downing, T., M.D., on Tic Douloureux and other painful Affections of the Nerves, with Suggestions for their Treatment by means of the Aneuralgicon, . . . . 386
Dental Surgery in Medical Colleges, on the Expediency and Impracticability of Teaching, . . . . 429

PAGE.
E
Experiments in the Use of Tin for Insertion of Teeth. By W. A. Royce, . . 9
Exposed Dental Pulp, Treatment of, . . . . . 35
Extraordinary Verdict, . 224
Exostosis, Observations on, 268
Epiglottis, Displacement of, 374
Extracting Teeth, a much less Painful and more Scientific Method of, . . . . 382
Ether and Chloroform, their Employment in Surgery, Dentistry, Midwifery, Therapeutics, &c. . . . . 390
Essay, Inaugural, on Zoo-Adynamia, . . . . . 390
Extracting Teeth, the Medical Students' Guide in, 388
Epulis, reported case of, By R. McLimont, D. D. S., 172
Elliot, W. A., D. D. S., Filling Teeth over Exposed Nerve, by, . . . 445
Ethics, Medical, . . 453
Epulis, . . . 561

F
Filling Teeth after the Pulp-Cavity has become Exposed, 12
File Carrier, a new, . 105
Filling Teeth after the Lining Membrane has become Exposed, . . . . 107
Files, Dental, . . 108
Fractures of the Jaw, on the Use of Gutta Percha in, 171
Filling Teeth, . . 182
Filling Teeth over Exposed Nerve, . . . . 445
Food and the Teeth, Observations on the Inorganic Constituents of the Food of Children, as connected with the Decay of the Teeth, . 192
Fractures of the Lower Jaw, Reinhardt's Adjuster for, 226
Forceps, Improved, . 227
Foil, Gold, amount used in the United States for Filling Teeth, . . 228
Filling Teeth, . 273, 478

PAGE.

Flemming, James, M. D., on Atmospheric Pressure, 308
Fatal Results from the Inhalation of Chloroform, . 315
Fistula Lachrymalis caused by Syphilis, . . 321
Fulcrum, Gilbert's, . 84
Flagg, J. F. B., M. D., Ether and Chloroform, their Employment, &c. . . 390

G

Gum Elastic Rings, Importance of Regulating the Teeth and Employment of, 28
Gilbert's Fulcrum, . 84
Gum Elastic, in the Treatment of Irregularity of the Teeth, 102
Gold for filling Teeth, a new Preparation of, . . 102
Gutta Percha, to Dental purposes, application of, . 145
Gutta Percha, in Fractures of Jaw, . . 171
Growth of Hair, Nails, and Secretions of the Body, . 225
Gold Foil, used in the United States, amount of, . 228
Gums in Phthisis, on the Appearance of the, . 372
Gardette, Dr. James, Biographical Notice of, . . 375
Gardette, Dr. E. B., on the Propriety of Abolishing Dental Colleges, and Substituting for them a Lectureship on Dentistry in Medical Schools, 398
Gilbert, H., M. R. C. S. L., &c. Method of Extracting Teeth, by, . . . 375
Gustatory Nerve, Division of the, . . . 545
Gardette, Dr., and his Proposition once more, . . 557

H

Hints on the Subject of Artificial Teeth and Plates, . 32
Hawes' Moulding Flask, 75
Handy, Prof. W. R., M. D., Relation between the Dental Organs and the Uterus, by, 127

PAGE.

Harris, Chapin A., M. D., Observations on the Etiology and Pathology of Odontatrophia, by, . . . 132
Hare-Lip, Observations on, 155
Holder, new Tongue, . 227
Hornor, S., the Medical Students' Guide in Extracting Teeth, by, . . 388
Hospital, Maryland, for the Insane, Reports for the year 1850, . . . 394
Hullihen, S. P., a new kind of Artificial Palate, with Observation on the Changes that take place in the Velum and Pharynx, during Deglutition, Respiration and Articulation, 245
Hemorrhage from the Tongue, treatment of, . . 501
Headache, &c., E. S. Ormerod, on, . . 505

I

Importance of Regulating the Teeth and Employment of Gum Elastic Rings in the Operation, . . 28
Journal, new series of the, 97
Journal of Health, N. Y. Med. Gazette, . . 106
Importance of the Preservation of the Teeth, . . 165
Inventors of Dental Instruments, Chairs, &c. . 226
Improved Forceps, . 227
Jourdain, M., . . 391
Journal, the, . . 404
Iodine in the Treatment of Dental Periostitis and Diseases of the Gums, by, . . 437
Johns, John A., M. D., on Morbid Sympathies of the Teeth, . . . 447
Jaw, Injury of, following the Removal of an Incisor, . 492

K

Kitsell, Jas., Esq., Displacement of the Epiglottis, by, 374

L

Lower Jaw, new mode of Reducing a Dislocation of, 90

PAGE,

Letter from Samuel Rambo, 105

Letter from Warren Rowell, 106

Lining Membrane, Filling Teeth after the, . . 107

Levett's Patent Enamel and its discovery, a test of, . 263

Lachrymalis, caused by Syphilis, Fistula, . . 321

Lecture on the Diseases of the Inferior Maxilla. By John Simon, Esq., Surgeon to St. Thomas' Hospital, . 332

Lecture, an Introductory. By Jno. Bell, M. D., to the Ohio Medical College, . 393

Lecture, an Introductory. By John Ware, M. D., to the Medical College, Mass., 303

Levison, J. S., Esq., Vis Nervosa from a Carious Tooth, 503

M

Moulding Flask, Hawes' 95
McLimont, R., D. D. S., case of Epulis, by, . . 173
Mechanical Dentistry, . 174
Mechanical Dentistry, . 305
Maxilla, Reproduction of, 311
Medicine and Surgery, in Egypt, 322
Maxilla, Diseases of, . 332
Microscopical Researches of the Teeth, . . 348
Macdonnell, R. M., M. D., of Montreal, Removal of the Upper Jaw, by, . 362
Maryland Hospital Reports, 394
Murphy, Jno. G., M. D., Review of Chemistry, for Students, . . . 390
Medical Ethics, . . 453
Maxillary, Necrosis of the Inferior, . . . 494

N

Ninth annual meeting of the American Society of Dental Surgeons, . . . . 36

New Mode of Reducing a Dislocation of the Lower Jaw, 90

New Series of the American Journal, . . . 10

New Drill Stock, . . 197

New Preparation of Gold for Filling Teeth, . . 102

PAGE.
Nomenclature of the Teeth, 104
New File Carrier, . 105
New York Register of Pharmacy and Medicine, . . 106
Nasal Polypus, . . 221
Necrosis from Phosphorous Vapor of the Inferior Maxilla, . . . 311
Nutz, H. K., Treatment of Exposed Pulp, by, . . 35
North, Jas., M. D., D. D. S., 437
Neuralgia, . . . 561

O

On the Use of a Tin as a Base for Artificial Teeth, . 3
On the Sensibility of the Teeth, 22
Ohio College of Dental Surgery 100
Obituaries, . . . 108
Observations on Hare-Lip, By Alfred A. Blandy, M. D. 155
Ovarian Cyst, a Dissection from the Membrane, taken from a Girl after Death, . 218
Observations on the Etiology and Pathology of Odontatrophia. By Chapin A. Harris, M. D. . . 132
Odontatrophia, Observations on. By Chapin A. Harris, M. D. . . . 132
Odontalgia, . . . 228
Omitted Notices, . . 228
On the Appearance of the Gums in Phthisis, . . 372
Ozena, . . . 487
Ormerod, E. L., on Headache, 505

P

Pulp Cavity, Filling after the Exposure of a, . . 12
Partial Deafness of twenty years standing, . . 89
Premium Teeth, . . 102
Percentage System among Dentists, . . . 151
Preservation of the Teeth. By L. S. Parmly, M. D. . 165
Parmly, L. S., M. D. . . 165
Paul, James, M. D., on the Food and Teeth, . . 192
Polypus Nasal, . . . 221
Progress of Dental Surgery, 222
Partner Wanted, . . 228

PAGE.
Pulp, Dental, Preparatory to Plugging. By J. D. White, 297
Periostities of the Upper Jaw, in connection with the Habit of Biting Coin, . . . 369
Phthisis, Appearance of the Gums, . . . . 372
Proceedings of the American Philosophical Society, April, December, 1850, . 394
Powders, Acidulated Tooth, 401
Parmly, Dr. Eleazar, Valedictory Address by, . . 460
Phrenology, . . 563

R

Royce, W. A., Experiments in the Use of Tin for Insertion of Teeth, . . . 9
Regulating Teeth, Employment of Gum Elastic Rings, 28
Report of the Committee of the American Society of Dental Surgeons, on the propriety of of Rescinding the Amalgam Pledge, . . . . . 66
Regulating Teeth by Spiral Springs, . . . . . 76
Reinhardt's Adjuster for fractures of the lower jaw, . 226
Reproduction of the Inferior Maxilla after Necrosis from Phosphorous Vapor, . 311
Removal of the Upper Jaw, &c. By Robt. M. McDonnell, M. D., . . . . . . 362
Review of Chemistry, by John G. Murphy, M. D. . . . 390
Record, M. on Chloroform, &c. 539
Rhinoplastic Operation, . 541

S

Spiral Springs, Regulating by, 76
Society, American, of Dental Surgeons, ninth Annual Meeting of, . . . . 36
Society, American, of Dental Surgeons, Report of Committee, &c. . . . . . 66
Society, American, of Dental Surgeons, . . . . 99
Superior Maxillary Bone, Removal of, under the influence of Chloroform, . . 312

PAGE.
Syphilis, Fistula Lachrymalis caused by, . . . 321
Surgery and Medicine in Egypt, 322
Simon, John, Esq., Surgeon to St. Thomas' Hospital.—A Lecture on the Diseases of the Inferior Maxilla, . . 332
Schwann, Dr. T., Microscropical Researches into the structure of the Teeth, . . 348
Success in the Medical Profession—an Introductory Lecture, delivered at the Mass. Med. College, by Jno. Ware, 393
Stockton's new style of Mineral Teeth, . . . . . 403
Skey, Mr. on the use of Chloroform, . . . . . 529
Sloughing Phagedena, &c. 532
Smith, J. V. C., M. D., 563

T

Tin for Insertion of Teeth—experiments in the use of, . 9
Touting System amongst Dentists, . . . . . 16
Teeth, sensibility of, &c. . 22
Transylvania School of Dental Surgery, . . . . 107
Teeth, Filling over exposed lining membrane, on, . . 107
Teeth Filling, by James Taylor, M. D., D. D. S. . . 182
Teeth, morbid sympathies of, 447
Tongue, new holder, . 227
Townsend, E., D. D. S., &c. Daily Jottings in the Gold Book of a Dentist, by, . 252
Taylor, Jas. M. D., on Filling Teeth, . . . 270, 478
Tumor, Cancerous, within the antrum, . . . . 312
Teeth, Skin and Hair, imperfect development of, &c. . 320
Townsend, Elisha, D. D. S., on Teaching Dental Surgery in Medical Colleges, , . 429
Teeth, Filling over Exposed Nerve, . . . . . 445
Teeth, Artificial, comparative advantages of wide and narrow plates for, . . . 458
Temporal Bone, diseased, . 499

PAGE.
Tongue, hemorrhage of, &c. 501
Teeth as an indication of the progressive improvement of the human race, . . 508
Tooth, Carious, Vis Nervosa, 503
Tongue, Cancer of, &c. . 545

W

White, J. D., Treatment of Dental Pulp, by, &c. . 297
Ware, John, M. D., Prof. of Theory and Practice in Harvard University—an Introductory Lecture, . . 393
Wheels, Corundum, . . 404

Z

Zeiderholz's Self-acting Blow Pipe and Lamp, . . 103
Zoo-Adynamia, an Inaugural Essay on, by Geo. J. Zeigler, M. D. . . . 390

